# Seasonal succession and niche differentiation in *Skeletonema* species driven by temperature and salinity in inner Tokyo Bay

**DOI:** 10.1111/jpy.70168

**Published:** 2026-04-24

**Authors:** Toshiya Katano, Tomohiro Suematsu, Ayane Tanaka, Saori Yasui‐Tamura, Fuminori Hashihama

**Affiliations:** ^1^ Department of Ocean Sciences Tokyo University of Marine Science and Technology Minato‐ku Tokyo Japan; ^2^ Department of Marine Environmental Science Tokyo University of Marine Science and Technology Minato‐ku Tokyo Japan; ^3^ Graduate School of Marine Science and Technology Tokyo University of Marine Science and Technology Minato‐ku Tokyo Japan; ^4^ Present address: Graduate School of Agriculture and Life Science The University of Tokyo Bunkyo Japan; ^5^ Present address: Tokyo Metropolitan Research Institute for Environmental Protection Koto Japan

**Keywords:** community organization, diatoms, niche differentiation, seasonal succession

## Abstract

The genus *Skeletonema* is a dominant diatom in coastal waters worldwide, frequently causing blooms, and it includes several cryptic species. To elucidate the occurrence patterns and niche differentiation among *Skeletonema* species in Tokyo Bay, Japan, sampling was conducted between June 2021 and February 2023. Species composition and abundance were determined using species‐specific quantitative polymerase chain reaction (qPCR) and were analyzed using an environmental framework in temperature, salinity, and nutrient concentrations. Niche characteristics were determined using outlying mean index (OMI) analysis, focusing on five frequently detected species: *S. ardens*, *S. marinoi–dohrnii* complex, *S. menzelii*, *S. potamos*, and *S. japonicum*. Seasonal succession was evident, wherein *S. japonicum* predominated in winter, followed by the *S. marinoi–dohrnii* complex in spring and *S. menzelii*, *S. ardens*, and *S. potamos* in summer. Principal component analysis indicated that *S. japonicum* was associated with low temperature and high salinity, whereas other frequently detected species were positively correlated with temperature. The OMI analysis revealed that niche breadth differed among species, with *S. potamos*, *S. marinoi–dohrnii* complex, and *S. menzelii* showing a wide niche breadth and *S. ardens* and *S. japonicum* being specialists. Marginality was highest for *S. potamos*, indicating an association with low salinity, but it was lowest for *S. japonicum* due to a high frequency of high‐salinity conditions. This study demonstrated that (1) seasonal succession in the bay is primarily structured by associations with temperature and salinity but (2) niche partitioning is not always clear. When the environment was highly disturbed in the summer rainy season, more than two *Skeletonema* species frequently co‐occurred, suggesting potential competition for nutrients.

**Abbreviations**
CCA: canonical correspondence analysis
CTD: conductivity temperature depth
DIN: dissolved inorganic nitrogen
DIP: dissolved inorganic phosphorus
DSi: dissolved silica
LSU: large subunit
OMI: outlying mean index
PCA: principal component analysis
qPCR: quantitative polymerase chain reaction
RDA: redundancy analysis
SEM: scanning electron microscopy
TEM: transmission electron microscopy
VIF: variance inflation factor

## INTRODUCTION

Diatoms contribute to approximately 20% of global primary production (Mann, 1999; Nelson et al., 1995) and often form large blooms in coastal waters (Carstensen et al., 2015), thus exerting a significant impact especially on coastal ecosystems. Although species of diatoms have long been identified and examined by light microscopy, species identification is sometimes insufficient because some of the species include cryptic species. The genus *Skeletonema* is a bloom‐forming diatom that is considered ecologically important due to its widespread distribution in coastal waters around the world, except in polar regions (Kooistra et al., 2008). Studies using electron microscopy and rDNA sequencing have revealed that *S. costatum* sensu lato (s.l.) comprises eight distinct species, and the genus currently consists of 12 species (Sarno et al., 2005, 2007; Zingone et al., 2005). Species composition has been explored in various regions (e.g., Canesi & Rynearson, 2016; Enjoji et al., 2019; Jung et al., 2009; Pfannkuchen et al., 2018; Yamada, 2013; Yamada et al., 2010, 2014; Yoshida et al., 2023; Yoshinaka et al., 2023), and studies have shown that multiple *Skeletonema* species co‐occur in the same waterbody, with different species forming blooms in different seasons. *Skeletonema* species exhibit different growth responses to temperature and salinity (Balzano et al., 2011; Kaeriyama et al., 2011). Therefore, species‐specific temporal monitoring is vital for understanding the bloom dynamics of *Skeletonema*.

Numerous studies have attempted to clarify seasonal succession among *Skeletonema* species (Canesi & Rynearson, 2016; Yoshida et al., 2023; Yoshinaka et al., 2023). For instance, Canesi and Rynearson (2016) quantitatively examined seasonal dynamics in Narragansett Bay using high‐throughput DNA sequencing based on LSU rDNA gene copy number. They determined that the winter–spring community, numerically dominated by *S. marinoi*, was markedly different from the summer–autumn community, which comprised multiple species, including *S. menzelii*, *S. pseudocostatum*, and *S. grethae/tropicum*. Moreover, Yoshida et al. (2023) explored the seasonal changing pattern of each *Skeletonema* species in the Ariake Sea, Japan, using quantitative polymerase chain reaction (qPCR) based on copy number and reported that *S. ardens* and *S. dohrnii*/*marinoi* occurred predominantly in winter, whereas *S. costatum* and *S. menzelii* were prevalent in summer. Because rDNA copy number varies among *Skeletonema* species (Canesi & Rynearson, 2016), Yoshida et al. (2023) did not determine the species composition (percentage cell density for each species to entire *Skeletonema*) among the *Skeletonema* species. In fact, cell density rather than gene copy number is necessary to examine the competitive relationship and/or niche differentiation among species. At a broader spatial scale, Yamada et al. (2022) documented the biogeography and ecological characteristics of *Skeletonema* species across multiple Japanese waterbodies, providing important context for species distributions. Although these studies have provided important insights into seasonal succession and species‐specific responses along individual environmental gradients, community‐level niche partitioning of *Skeletonema* species has rarely been examined using a multivariate framework that simultaneously incorporates temperature, salinity, and nutrient conditions.

In Tokyo Bay, *Skeletonema costatum* (s.l.) forms blooms throughout the year and accounts for 28%–98% of the total phytoplankton cell abundance (Han et al., 1992; Ueno et al., 2023; Yoshida & Ishimaru, 2008). Studies have confirmed the presence of multiple *Skeletonema* species in the bay (Jiang et al., 2024; Yamada et al., 2014; Yoshinaka et al., 2023). For instance, Yoshinaka et al. (2023) demonstrated that there existed a bimodal seasonal changing pattern of *Skeletonema* abundance, and these two peaks consist of different species, wherein winter/spring peak contained *S. japonicum* and the *S. marinoi–dohrnii* complex, and summer/autumn peak contained *S. potamos*, *S. ardens*, and *S. costatum* s.s. Nevertheless, their abundances at species level have not yet been clarified.

In the present study, we conducted quantitative, species‐specific monitoring of *Skeletonema* in Tokyo Bay using real‐time PCR to determine the cell densities. Our objectives were to elucidate the seasonal dynamics in abundance and investigate detailed niche partitioning among species with respect to temperature, salinity, and nutrient concentrations. We applied real‐time PCR because it enables the rapid and simple quantification of species, even within taxonomically challenging groups, through the use of species‐specific primers.

## MATERIALS AND METHODS

### Sampling

**FIGURE 1:**
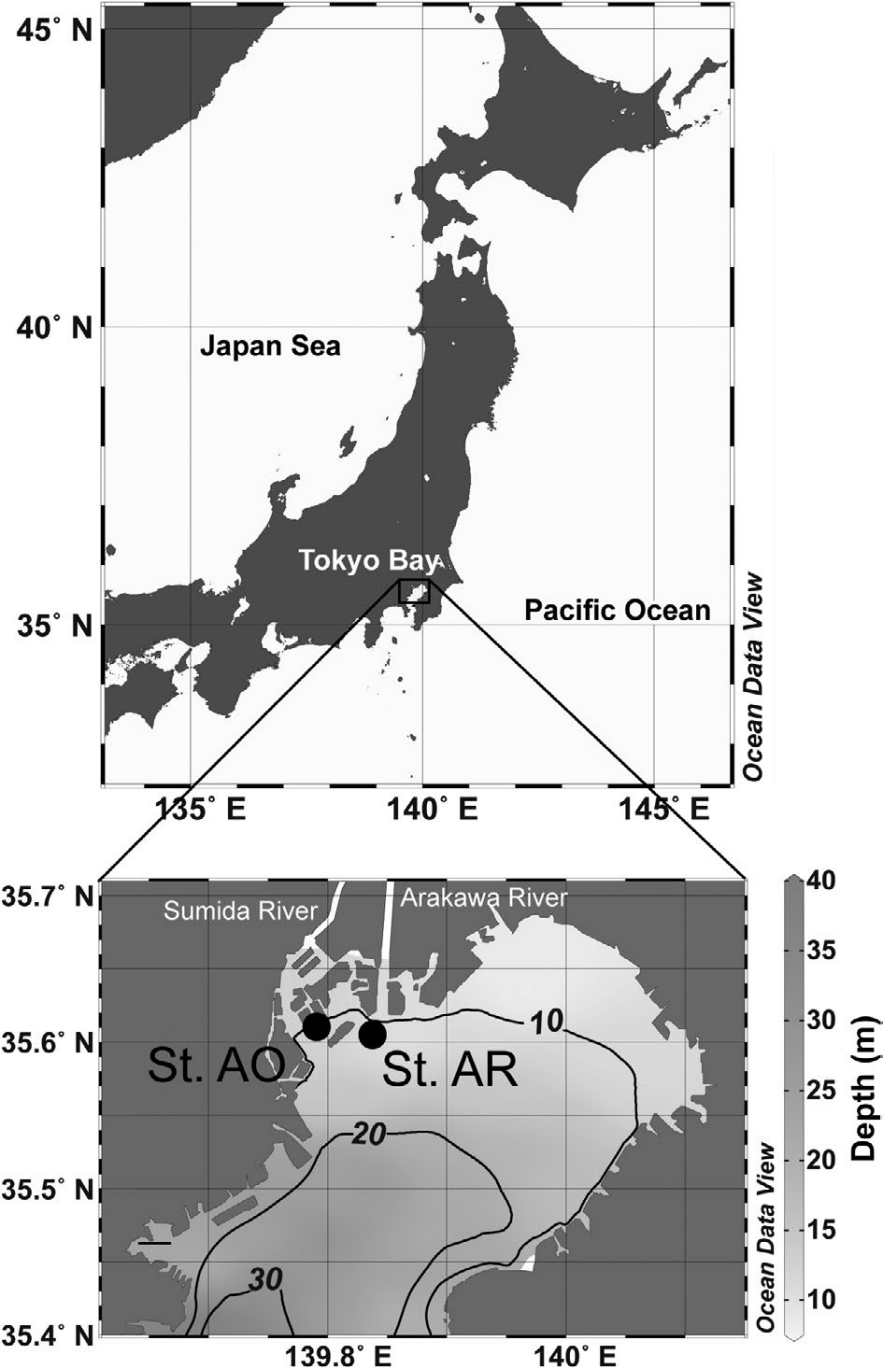
Map of sampling stations. Sampling stations are indicated by station codes: AO (Aomi) and AR (Arakawa).

Sampling was conducted approximately once a month from June 2021 to February 2023 in the inner part of Tokyo Bay (Stations Arakawa, St. AR, and Aomi, St. AO) using the training boat Hiyodori of Tokyo University of Marine Science and Technology (Figure 1). At St. AO, salinity tends to be lower when water temperature is higher, and vice versa, resulting in strong covariance between these two variables (Yoshinaka et al., 2023). Station AR, which is more strongly influenced by freshwater from the Arakawa River (Figure 1), was particularly useful for disentangling the effects of temperature and salinity. Data from both stations were therefore included in the analysis. Water temperature, salinity, and chlorophyll fluorescence were vertically measured using a conductivity temperature depth (CTD) instrument (JFE Advantech AAQ1183‐PRO), and water samples were collected using a bucket from the surface layer (depth 0 m) and using a Van Dorn sampler from the depth where chlorophyll fluorescence was at its maximum (hereafter referred to as “Peak”). A portion of the collected water samples was fixed using HEPES‐buffered paraformaldehyde and glutaraldehyde (Katano et al., 2009). The cell density of *Skeletonema* was then evaluated under a light microscope using either a plankton counting chamber (MPC‐200, Matsunami Glass) or a Fuchs‐Rosenthal hemocytometer (AS ONE, Osaka, Japan).

### Enumeration of *Skeletonema* species using real‐time PCR

First, 100–400 mL of the water samples were filtered using a 2.0‐μm polycarbonate membrane filter (47 mm in diameter; GVS Track Etched Membrane). DNA was then extracted using NucleoSpin® Plant II Kit (TaKaRa) according to the manufacturer's instructions. Real‐time PCR was conducted using LightCycler® 96 (Roche), with FastStart Essential DNA Green Master (Roche), using a species‐specific primer set designed by Enjoji et al. (2019). The PCR conditions were those described by Enjoji et al. (2019), with the exception that the template DNA volume was set to 1 μL, and the volume of water was adjusted to 7 μL. The volume of template DNA was reduced from 5 μL to 1 μL based on preliminary tests showing stable and reproducible amplification with smaller template volumes. The target species included the following nine *Skeletonema* species recorded in Japan: *S. ardens*, *S. costatum* sensu stricto (hereafter *S. costatum*), *S. grevillei*, *S. japonicum*, *S. marinoi–dohrnii* complex, *S. menzelii*, *S. pseudocostatum*, *S. potamos*, and *S. tropicum*.

Approximately 10^6^ or 10^7^ cells from each strain (Table S1)—*Skeletonema ardens* (TKC092), *S. costatum* (TKC103), *S. grevillei* (FON073), *S. japonicum* (TKC033), *S. dohrnii* (FDK033), *S. menzelii* (DM08090730), *S. pseudocostatum* (FDK225), *S. potamos* (P10#20), and *S. tropicum* (TKC156)—were collected onto 2‐μm polycarbonate filters, and DNA was extracted using NucleoSpin® Plant II Kit (TaKaRa) as mentioned above. Cell counting was conducted using light microscopy under 200× magnification. A 4‐ or 5‐step 10‐fold serial dilution of the extracted DNA was prepared and used as templates for the real‐time PCR. Species‐specific calibration curves were constructed by relating quantification cycle (Cq) values to known cell densities for each species (Table S2). Cell densities in water samples were then calculated by converting Cq values to cell densities using the calibration curves and by the filtered water volume.

### Measurement of chlorophyll *a* concentration

The concentration of chlorophyll *a* was measured fluorometrically (Welschmeyer, 1994). Briefly, a 50‐mL sample of seawater was filtered through a glass fiber filter (GF/F filter, Whatman), and chlorophyll *a* was extracted using 5 mL of N, N‐dimethylformamide (DMF) at −25°C under dark conditions (Suzuki & Ishimaru, 1990). The concentration of chlorophyll *a* was then determined using a fluorometer (10–AU Fluorometer, Turner Designs).

### Measurement of nutrient concentration

For the measurement of nutrient concentrations (DIN, nitrate + nitrite + ammonium; DIP, phosphate; and DSi, silicic acid), a portion of water samples was filtered using a polytetrafluoroethylene filter (DISMIC–25HP, pore size 0.2 μm, ADVANTEC) and frozen at −25°C for subsequent analysis. After thawing, the concentrations of nitrate, nitrite, ammonium, phosphate, and silicic acid were measured using standard spectrophotometric methods as described by Kubo et al. (2019) and Jiang et al. (2024) using an autoanalyzer (QuAAtro‐Marine 5 ch., SEAL Analytical), with the detection limits being 0.10, 0.02, 0.15, 0.05, and 0.04 μM, respectively.

### Data analysis

The composition of *Skeletonema* community was analyzed using biotic and abiotic environmental variables of water temperature, salinity, DIN, DIP, and DSi concentrations. For DIN, we separately analyzed the composition using ammonium concentration (NH_4_) and nitrate+nitrite concentration (NO_3_ + NO_2_).

For the niche analysis, the cell density (cells · mL^−1^) of each *Skeletonema* species was treated independently as the response variable. Relative abundance or percent contribution to the total *Skeletonema* community was used only to describe species composition and seasonal succession and was not used in the niche analysis. Pearson's correlation analysis was conducted on square‐root‐transformed percentage contribution data.

We next quantified the environmental niche position and breadth of *Skeletonema* species. The environmental dataset contained temperature (°C), salinity, and NH_4_, NO_3_ + NO_2_, DIP, and silicic acid (DSi) concentrations. To reduce multicollinearity among environmental variables, we calculated the variance inflation factor (VIF). The VIFs for the abovementioned factors were <10; hence, all factors were used in all subsequent analyses. Five frequently detected *Skeletonema* taxa, including *S. ardens*, *S. menzelii*, *S. marinoi–dohrnii* complex, *S. japonicum*, and *S. potamos*, were used for the analysis.

To explore major environmental gradients and general patterns of species–environment associations, principal component analysis (PCA) was applied as an exploratory analysis. The PCA was conducted using standardized environmental variables (temperature, salinity, and nutrient concentrations) together with species occurrence information to visualize seasonal and environmental trends among *Skeletonema* species. To quantitatively evaluate species‐specific niche characteristics, Outlying Mean Index (OMI) analysis was employed, which explicitly links species occurrences to environmental space and provides metrics of marginality (deviation from the mean environmental conditions of all samples) and tolerance (niche breadth). Because the primary objective of the present study was to characterize realized niches and niche differentiation among *Skeletonema* species rather than to model species abundance as a direct function of environmental variables, OMI analysis was considered more appropriate than constrained ordination methods such as redundancy analysis (RDA) or canonical correspondence analysis (CCA). All multivariate analyses were conducted in R using the ade4 package.

To quantitatively evaluate species‐specific niche characteristics beyond exploratory visualization by PCA, we computed the marginality and tolerance of the species according to the OMI framework (Dolédec et al., 2000) as implemented in the niche method of the ade4 package (R environment). Marginality (OMI) was defined as the squared Euclidean distance between the global mean environment (the centroid of all sample scores in PCA space) and the centroid of the species, in which the centroid was calculated as the weighted mean of the sample scores. Tolerance was measured as the weighted variance of the sample scores around the centroid of the species in PCA space. This metric represents the breadth of the environmental conditions used by the species. To determine the realized niches of each species, the centroids of species in PCA space were backtransformed into the original environmental units using the inverse PCA transformation and the original mean and standard deviation of the retained environmental variables.

## RESULTS

### Environmental condition, chlorophyll *a* concentration, and *Skeletonema* cell density determined by light microscopy

**FIGURE 2:**
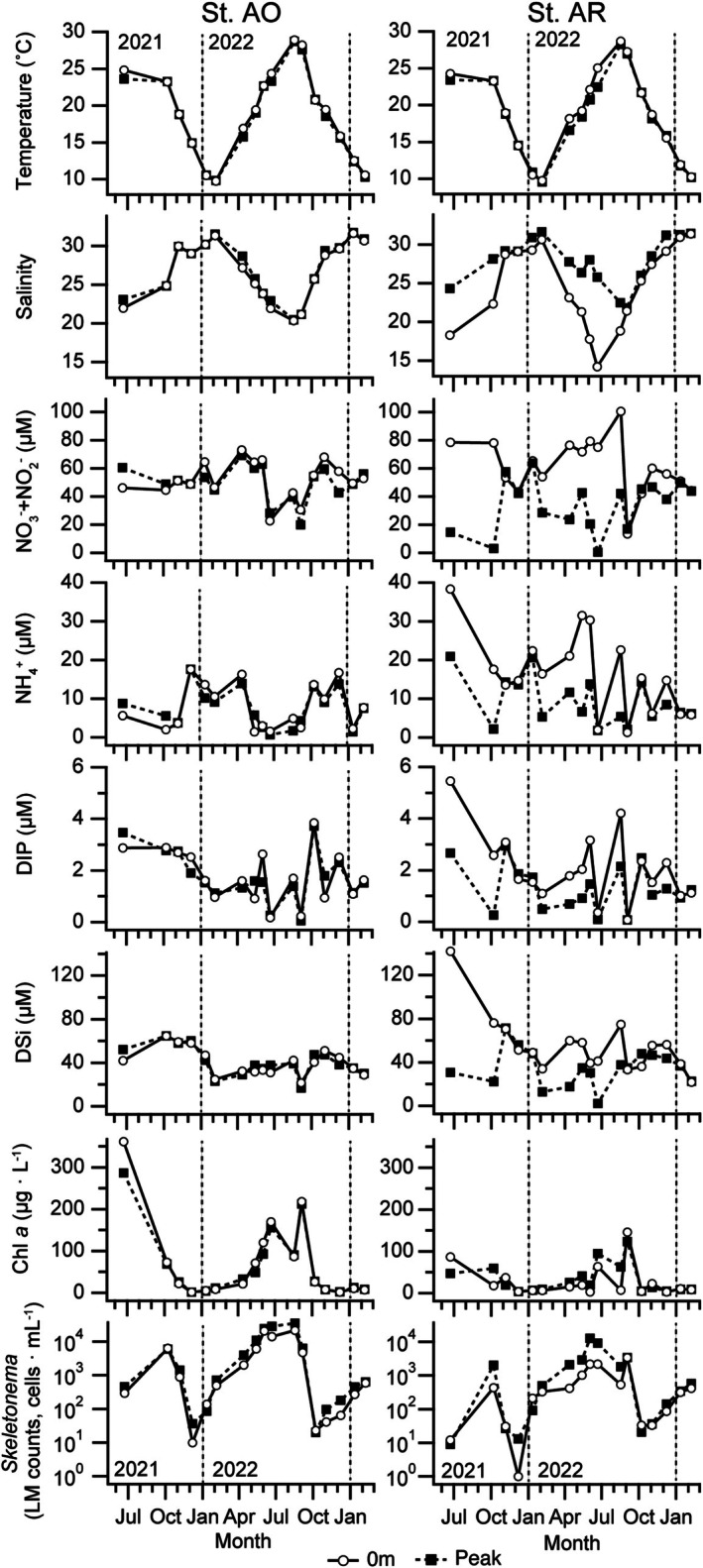
Changes in temperature and salinity and NO_2_ + NO_3_, NH_4_, DIP, DSi, and chlorophyll *a* concentrations and *Skeletonema* cell density determined by light microscopy at Sts. AO and AR.

Water temperature ranged between 9.6 and 28.9°C during the study period for the two stations, Sts. AR and AO (Figure 2). No difference in temperature was observed between the two stations, and the highest and lowest temperatures were recorded in August and February, respectively. Regarding salinity, it became low in the rainy summer seasons (<25) between July and September/October. At St. AR, the salinity was lower than that at St. AO, as the station was located near the mouth of Arakawa River (Figure 1). The lowest salinity of 14.2 was recorded at the surface in June 2022 (Figure 2). At the chlorophyll peak depth of St. AR, the salinity was not so low, and the value was similar to that at St. AO.

The concentrations of NO_3_ + NO_2_ were high (>40 μM) in most cases at St. AO and at the surface at St. AR (Figure 2). At the peak depth of St. AR, the concentrations of NO_3_ + NO_2_ were sometimes low, reaching <2 μM (October 2021 and June 2022). The NO_2_ concentration was generally low, with the mean and maximum concentrations being 3.86 and 6.99 μM, respectively (data not shown). The NH_4_ concentration periodically fluctuated at St. AR. In the winter season, a relatively high NH_4_ concentration was observed (>10 μM), but it became low (<10 μM) during the summer season at St. AO. The DIP concentrations were relatively high in both layers of St. AO and surface of St. AR. Nevertheless, occasionally low DIP concentrations were detected in the summer season. The DSi concentration was relatively and constantly high (>20 μM) at St. AO. At St. AR, a quite high DSi concentration (>120 μM) was detected in July 2021. At the peak depth, the DSi concentration was constantly high (>15 μM) with an exception at the peak depth of St. AR in June 2022.

Quite high chlorophyll *a* concentrations (>280 μg · L^−1^) were recorded in June 2011 at St. AO. Similarly, high chlorophyll *a* values (>85 μg · L^−1^) were also recorded between June and September 2022 at St. AO (Figure 2). In the winter season, chlorophyll *a* concentrations became low, with the lowest value (2.03 μg · L^−1^) recorded in December 2021 at St. AO.

*Skeletonema* counts determined under a light microscope ranged from 0 to 35,624 cells · mL^−1^. In general, the cell density was high between May and September (>10^3^ cells · mL^−1^) and low (<10^3^ cells · mL^−1^) between November and February (Figure 2).

### Real‐time PCR assays

We generated calibration curves for the nine species in each experiment. The correlation coefficients and efficiencies for each species were 1.00 and 1.76–1.96 for *Skeletonema ardens*, 1.00 and 1.76–1.96 for *S. costatum*, 1.00 and 1.82 for *S. grevillei*, 1.00 and 1.71–1.86 for *S. japonicum*, 0.99–1.00 and 1.70–1.85 for *S. marinoi–dohnrii* complex, 0.99–1.00 and 1.72–1.91 for *S. menzelii*, 1.00 and 1.83 for *S. peseudocosutatum*, 0.99–1.00 and 1.73–1.91 for *S. potamos*, 1.00 and 1.83 for *S. tropicum*, respectively.

We compared the cell densities enumerated by light microscopy and real‐time PCR assay and observed that the total cell counts from real‐time PCR assay for the nine species did not correspond well with the counts determined by light microscopy (*R* ^2^ = 0.85, *F*_1, 66_ = 384.7, *p* < 0.01, Figure S1). Nevertheless, the results of real‐time PCR assay were highly consistent with those of light microscopy when *Skeletonema potamos* and *S. menzelii* were excluded. *Skeletonema menzelii* typically occurs as solitary cells in nature (Sarno et al., 2005), and *S. potamos* frequently forms two‐cell colonies (Torgan et al., 2009). In addition, both species have relatively small cell sizes and atypical morphologies, which may reduce their detectability in routine light microscopy. Therefore, these two species may have been underestimated or overlooked under the light microscope.

### *Skeletonema* species composition, seasonal succession, and niche differentiation

Among the nine *Skeletonema* species we examined, *S. ardens* was the most frequently detected, occurring in 64 of 68 samples (Table 1), followed by the *S. marinoi–dohrnii* complex (62), *S. menzelii* (60), *S. potamos* (56), *S. japonicum* (43), and *S. costatum* (20). In the present study, the term “dominant species” is used to refer to species that were both frequently detected and relatively abundant in terms of cell density. The remaining three species were rarely detected at either station. Therefore, we considered the first five species as dominant in the study area.

**TABLE 1:** Occurrences and percentage contributions of each species to the entire *Skeletonema* community.

Species	Number of samples^a^	Mean cell density (cells · mL^−1^)	Mean percentage (%)	Maximum cell density (cells · mL^−1^)	Maximum percentage (%)
*S. ardens*	64	1069.36	12.81	28,775.00	82.23
*S. marinoi–dohrnii* complex	62	1686.07	20.20	18,620.00	99.22
*S. menzelii*	60	2787.47	33.40	58,190.00	76.20
*S. potamos*	56	2652.59	31.78	121,500.00	97.17
*S. japonicum*	43	123.75	1.48	976.00	94.72
*S. costatum*	20	26.43	0.32	493.30	3.83
*S. tropicum*	2	0.09	0.00	3.86	0.08
*S. grevillei*	1	0.21	0.00	12.35	1.96
*S. pseudocostatum*	0	0.00	0.00	0.03	0.00

^a^ Total number of samples was 68.

The mean cell densities were highest for *Skeletonema menzelii* and *S. potamos*, both exceeding 2000 cells · mL^−1^, followed by *S. ardens* and the *S. marinoi–dohrnii* complex. *Skeletonema japonicum* and *S. costatum* showed lower mean densities (<1000 cells · mL^−1^, Table 1). Similarly, the mean percentage contribution to the total *Skeletonema* community was highest for *S. potamos* and *S. menzelii*, followed by the *S. marinoi–dohrnii* complex and *S. ardens*. *Skeletonema japonicum* and *S. costatum* each contributed <2% on average, despite *S. japonicum* being frequently detected. The maximum percentage contributions exceeded 76.2% for all the five dominant species, and remarkably, *S. japonicum* constituted 94.72% despite its low mean contribution.

**FIGURE 3:**
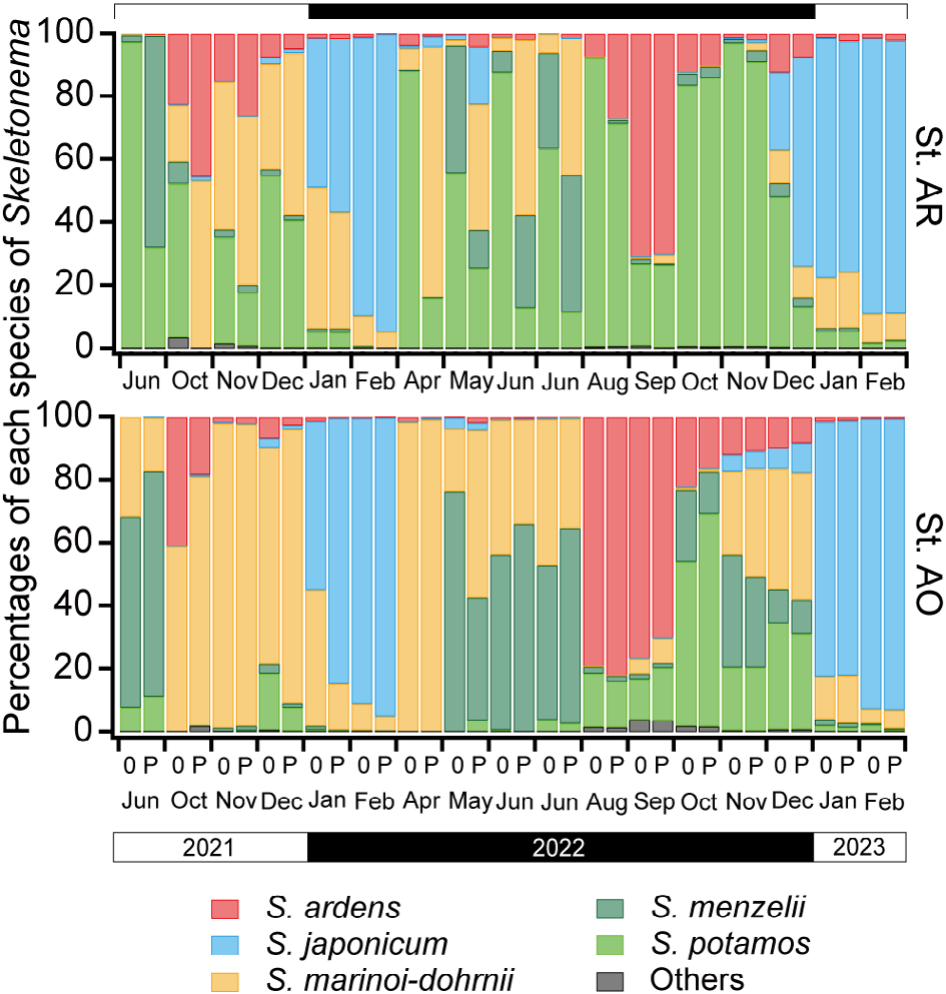
Changes in the species composition of *Skeletonema* at Sts. AO and AR. In the horizontal axis, 0 and P indicate the surface and the chlorophyll peak depth, respectively.

Seasonal succession was evident (Figure 3). In January and February, *Skeletonema japonicum* predominated, accounting for >60% of the total *Skeletonema* cell density (Figure 3). In April, the community shifted to dominance by the *S. marinoi–dohrnii* complex in the deeper layers at both stations. In May and June, *S. menzelii* co‐occurred with the *S. marinoi–dohrnii* complex. In August at St. AO, the community rapidly shifted to dominance by *S. ardens*, with a similar shift occurring at St. AR in September. The *S. ardens*‐dominated community gradually transitioned to a mixed assemblage of *S. potamos* and *S. menzelii*. At St. AO, the *S. marinoi–dohrnii* complex reappeared in November and December.

**FIGURE 4:**
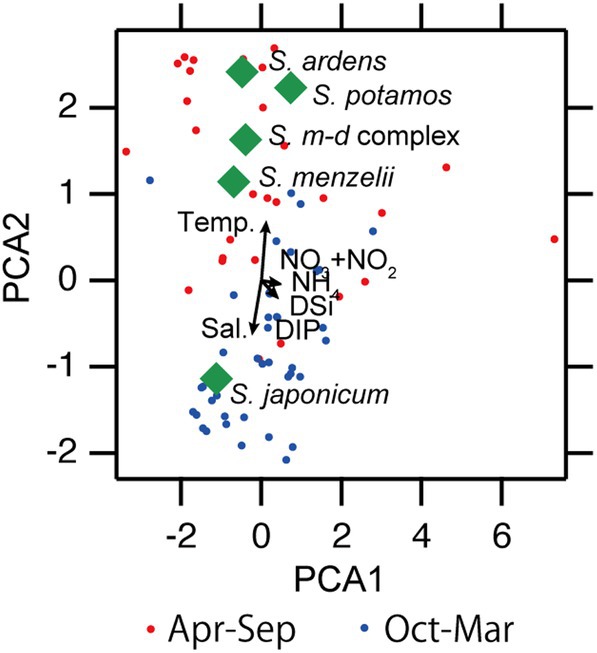
Principal component analysis (PCA) plots depicting the relationships between *Skeletonema* species, environmental variables, and community samples in the present study.

Principal component analysis was used to visualize major environmental gradients and general species–environment associations (Figure 4). The first two principal components primarily reflected variation in temperature and salinity. Among the *Skeletonema* species, *S. japonicum* was clearly associated with low temperature and high salinity, whereas the other frequently detected species showed positive associations with temperature. No clear separation of species along nutrient gradients was observed in the PCA space. Because the PCA did not reveal strong nutrient‐driven separation among species, niche differentiation was further quantified using OMI analysis, which explicitly incorporates the distribution of environmental conditions across all samples.

**FIGURE 5:**
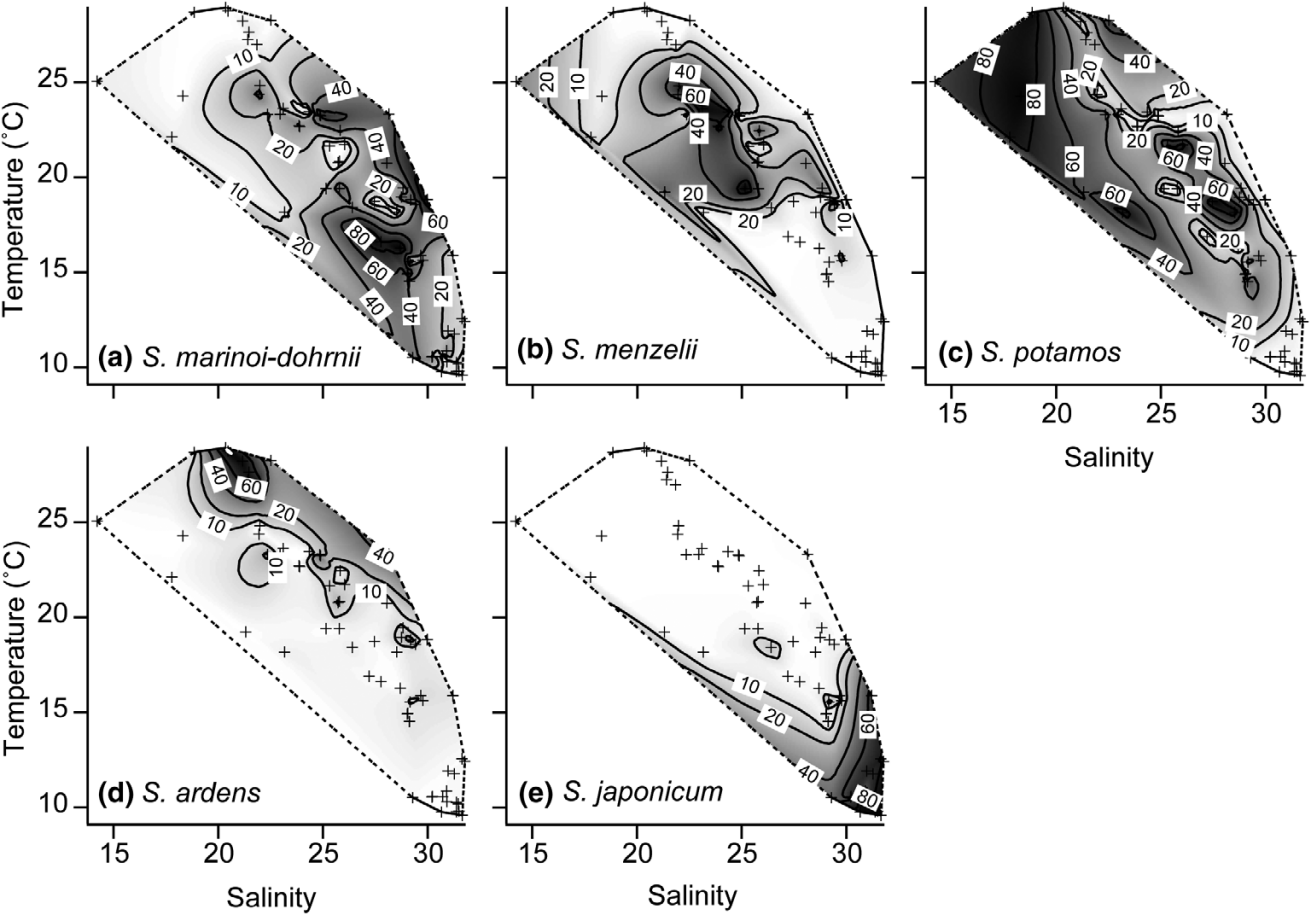
Contour plots for the percentage contribution of *Skeletonema* species to the entire community in relation to temperature and salinity.

Because species occurrence appeared to be more strongly influenced by temperature and salinity than by nutrients, we focused on these two variables to describe the niches of the dominant species (Figure 5). We observed that the *Skeletonema marinoi–dohrnii* complex occurred across a wide range of salinities and temperatures, with the highest percentage contribution (~80%) at salinities of 25–28 and temperatures of 16–18°C. A secondary peak occurred at salinities of 28–29 and temperatures of 18–22°C. The percentages decreased to <10% when salinity was <20. *Skeletonema menzelii* also occurred under intermediate conditions, dominating at salinities of 21–25 and temperatures of 18–25°C, although the percentage contributions generally did not exceed 60% (maximum 76.2%, Table 1). *Skeletonema potamos* predominated when salinity was <20 (Figure 5), outcompeting all other species (maximum 87.17%, Table 1); however, it also occurred at salinities of >20, with contributions exceeding 60% at salinities of 24–27 and temperatures of 18–21°C. *Skeletonema ardens* was associated with temperatures of >20°C (Figure 5), reaching its highest contribution (82.23%, Table 1) in waters at >25°C. *Skeletonema japonicum* occurred when salinity was >30 and temperature was <15°C (Figure 5), contributing up to 94.7% to the total *Skeletonema* community (Table 1).

**FIGURE 6:**
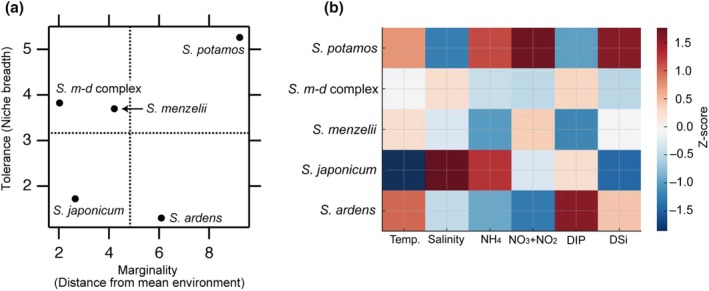
Panel a: Tolerance (niche breadth) and marginality (distance from the average environmental conditions of all samples) of *Skeletonema* species determined using outlying mean index (OMI) analysis. Panel b: Heat map of realized niches of the dominant species of *Skeletonema*. Colors represent standardized values (*z*‐scores) of each species for each environmental variable. Positive values (red) indicate association with conditions higher than the mean of centroids of all species, and negative values (blue) indicate association with conditions lower than the mean.

The niches of *Skeletonema ardens* and *S. japonicum* were clearly distinct, with *S. ardens* occupying warm, high‐salinity conditions, whereas *S. japonicum* predominated under cold, high‐salinity conditions (Figure 5). These species rarely co‐occurred with other species (Figure S2). *Skeletonema menzelii* and the *S. marinoi–dohrnii* complex frequently co‐occurred (Figures 3 and 4, Figure S2), with a significant positive correlation in the percentage contribution (*r* = 0.87, *t* = 14.37, *df* = 66, *p* < 0.01). These data indicate that niche partitioning is not always established among *Skeletonema* species, as the three dominant species (*S. marinoi–dohrnii* complex, *S. menzelii*, and *S. potamos*) exhibited relatively wide niche breadths (Figure 6).

Niche breadth was highest for *Skeletonema potamos*, followed by the *S. marinoi–dohrnii* complex and *S. menzelii* (Figure 6). *Skeletonema potamos* predominated when salinity was <20 but also occurred across a wide salinity–temperature range (Figure 5), resulting in the highest tolerance and marginality among species (Figure 6). The *S. marinoi–dohrnii* complex and *S. menzelii* also exhibited wide niches (Figure 5), although marginality was slightly lower for the former due to its occurrence in both spring (April) and autumn (November; Figures 3 and 6). *Skeletonema menzelii* occurred in early summer (Figure 3) and in relatively low‐salinity conditions (Figure 5), resulting in slightly higher marginality than that of the *S. marinoi–dohrnii* complex (Figure 6). The remaining two species, *S. japonicum* and *S. ardens*, were specialists (Figure 6).

Finally, we examined the favorable conditions for each dominant species (Figure 6). We observed that the generalist *Skeletonema potamos* was associated with low salinity and high temperature and it tended to cooccur with relatively higher inorganic nitrogen and DSi concentrations. In contrast, the *S. marinoi–dohrnii* complex, another generalist, showed no clear environmental association and occurred under diverse conditions. *Skeletonema menzelii* also exhibited no clear association, although DIP concentration showed a weak negative correlation with its occurrence. *S. japonicum* demonstrated a strong negative relationship with temperature and a positive relationship with salinity and was weakly associated with higher NH_4_^+^ concentrations and lower DSi concentrations. *Skeletonema ardens* occurred under high‐temperature and high‐salinity conditions, and was occasionally associated with relatively lower DIN and higher DIP concentrations.

## DISCUSSION

More than one species of phytoplankton within a genus frequently co‐occurs in the same waterbody (Needham & Fuhrman, 2017; Trombetta et al., 2021; Vineis et al., 2011). In the case of toxic and/or harmful species, species‐rank analysis is essential for ecosystem management and fisheries. Nevertheless, such analyses are not always conducted sufficiently. In addition to the genus *Skeletonema*, several diatom genera such as *Pseudo‐nitzschia*, *Thalassiosira*, and *Chaetoceros*, as well as the dinoflagellate genus *Alexandrium*, include cryptic or pseudocryptic species (Arin et al., 2022; Fehling et al., 2006; Malviya et al., 2016). Accurate identification of these species generally requires detailed observation of fine structures by transmission (TEM) and/or SEM, combined with molecular analyses. Therefore, species‐rank identification and enumeration from natural samples have long been challenging, and consequently, niche partitioning among these species remains poorly understood.

Among these diatom genera, *Skeletonema* represents one of the most ecologically significant and taxonomically complex groups in coastal ecosystems and frequently forms blooms in both winter and summer seasons (Ueno et al., 2023). In the present study, we successfully examined the species‐rank dynamics at two stations in Tokyo Bay and demonstrated niche partitioning among five *Skeletonema* species using species‐specific qPCR. We also observed that the ecological characteristics of each species differed not only in their niche position but also in their niche breadth. We believe that this approach will contribute to a better understanding of the mechanisms underlying competition and co‐occurrence among closely related species in natural environments.

### Seasonal occurrence pattern

The seasonal pattern of *Skeletonema* cell density observed in our study conducted between 2021 and 2023 (Figure 2) differed from that reported previously for St. AO, which was studied between 2016 and 2020 (Yoshinaka et al., 2023). In Yoshinaka et al. (2023), a bimodal pattern was detected, with a first peak between February and May and a second peak between July and October, and cell densities decreasing to <10^2^ cells · mL^−1^ between the two peaks. In contrast, in the present study, no clear bimodal pattern was detected. Instead, low densities (<10^2^ cells · mL^−1^) occurred between October and December at both stations, followed by a gradual increase, resulting in a single summer peak between June and August (Figure 2). The absence of the early summer minimum in 2019 (Yoshinaka et al., 2023) may reflect interannual variability in seasonal abundance patterns after 2019, which warrants further investigation.

Seasonal variation in the species richness of *Skeletonema* in Tokyo Bay resembled that in Narragansett Bay, with low richness (fewer than two species) in winter and higher richness (fewer than six species) in warmer months. This pattern is consistent with reports from other coastal systems, where *S. japonicum* often peaks in winter, as observed in the Ariake Sea (Yoshida et al., 2023) and in December 2012 in Narragansett Bay (Canesi & Rynearson, 2016). This species exhibited a relatively high growth rate (>0.6, <1.5 · day^−1^) at 10°C, whereas other species, such as *S. tropicum* and *S. ardens* could not grow (0 · day^−1^) at this temperature (Kaeriyama et al., 2011). In most years in Narragansett Bay, *S. marinoi* was also the predominant winter species, showing a high growth rate of 0.61 · day^−1^ at 4°C (Anderson & Rynearson, 2020). Experimental studies further indicate that, among eurythermal *Skeletonema* species with similar temperature–growth responses, species with low thermal minima, such as *S. marinoi*, can dominate under low‐temperature conditions (Anderson & Rynearson, 2020).

From February to May, as the temperature increased and salinity decreased (Figure 2), the *Skeletonema* community shifted from *S. japonicum* to dominance by the *S. marinoi–dohrnii* complex in April, probably facilitated by its presence in the previous month (Figure 3). At St. AO, *S. menzelii* replaced the *S. marinoi–dohrnii* complex in May (Figure 3), consistent with its association for warm water and salinities of 18–36 (Guillard et al., 1974). Kaeriyama et al. (2011) also reported high growth (>2.5 · day^−1^) rates of *S. menzelii* at 35°C. At St. AR, *S. potamos* also became abundant, particularly in surface waters near the Arakawa River mouth (Figure 3), probably benefiting from reduced salinity during the rainy season (May–June, Figure 2). Although often considered a freshwater species (Duleba et al., 2014; Weber, 1970), *S. potamos* can grow at salinities of 2–24 (Paasche, 1975), making these conditions favorable.

From August onward, *Skeletonema ardens* predominated at St. AO, with the shift occurring later at St. AR (Figure 3). This dominance persisted through September. *Skeletonema ardens* has also been reported from tropical and subtropical waters such as Xiamen Harbor (China), Singapore, and the Gulf of Carpentaria (Australia; Gu et al., 2012; Sarno et al., 2007). Kaeriyama et al. (2011) reported that *S. ardens* grows rapidly (>2.0 · day^−1^) even at 35°C. This species probably thrives in the warm summer conditions of Tokyo Bay. In November and December, the diversity of *Skeletonema* increased, whereas the total cell density decreased to <10^3^ cells · mL^−1^ (Figure 2).

### Niche partitioning in Tokyo Bay

Traditional niche theory predicts that closely related species are less likely to coexist due to competitive exclusion, assuming that phylogenetic relatedness reflects ecological similarity (Elliot & Davis, 2017). The seasonal succession observed in Tokyo Bay suggests that species differentiation within *Skeletonema* has resulted in distinct growth characteristics as summarized in Table 2. *Skeletonema japonicum* and *S. ardens* obviously occupied distinct environmental niches compared with other *Skeletonema* species. Nonetheless, niche partitioning was not always evident. *Skeletonema menzelii* frequently co‐occurred with the *S. marinoi–dohrnii* complex at St. AO and with *S. potamos* at St. AR, and *S. potamos* often co‐occurred with both species (Figure 3, Figure S2). For these three species, niche partitioning was unclear, which is consistent with their relatively high tolerance values from the OMI analysis (Figure 6).

**TABLE 2:** Niche of *Skeletonema* species in Tokyo Bay.

Species	Temperature	Salinity	Nutrients	Niche in TB
*S. japonicum*	<15	>28	High NH_4_ and low DSi concentrations	Low‐temperature specialist
*S. marinoi–dohrnii* complex	<29	25–28	Low to moderate concentrations	Generalist
*S. ardens*	>25	20–25	Low N and high DIP concentrations	High‐temperature specialist
*S. menzelii*	20–25	20–26	Low DIP and NH_4_ concentrations	Generalist
*S. potamos*	>15	<28	High N and DSi concentrations	Generalist (Low salinity, High NSi)
*S. costatum*	–	–	–	Minor species

*Note*: Nutrient variables are included for completeness; however, their associations were generally weaker than those of temperature and salinity and are interpreted as secondary.

The lack of strict niche separation among the *Skeletonema marinoi–dohrnii* complex, *S. menzelii*, and *S. potamos* (Figures 3, 5 and 6) suggests that seasonal succession among them is affected more by fluctuating nutrient availability than by temperature or salinity alone. In fact, *z‐*score analyses of environmental associations suggested that *S. potamos* dominance often coincided with higher inorganic nitrogen and DSi concentrations, whereas *S. menzelii* tended to occur under lower DIP concentrations (Figure 6). However, because nutrient concentrations are not fully independent variables and may be reduced as a result of phytoplankton uptake, these associations should be interpreted with caution and do not necessarily indicate direct nutrient limitation. Taken together, these findings emphasize the potential role of nutrient stoichiometry in structuring *Skeletonema* assemblages, a factor that should be investigated in future experimental and field studies.

*Skeletonema potamos* is a facultative freshwater species in river ecosystems worldwide, primarily temperate zones, such as the Danube River in Europe, the Missouri River in the United States (Duleba et al., 2014), and the Seta River in Japan (Lake Biwa Environmental Research Institute, 2022). It was also detected in San Francisco Bay Estuary when the water temperature and salinity were high (Lehman, 2000) as well as in the Ariake Sea (Enjoji et al., 2019; Yoshida et al., 2023). Although the life history of this species is currently not well understood, it may proliferate in estuarine waters when river discharge increases during the warm season.

## CONCLUSIONS

Seasonal succession and niche partitioning among cryptic species of *Skeletonema* in coastal waters have long been important but are unresolved topics in phycology. The present study demonstrated a clear seasonal succession pattern in Tokyo Bay, one of the most highly eutrophic embayments, using species‐specific qPCR assays. As anticipated, we identified distinct ecological niches for some species, such as *Skeletonema japonicum* and *S. ardens*. Nevertheless, we also observed that several species demonstrated wide niche breadths with a substantial overlap, indicating that interspecific competition still occurs within the genus *Skeletonema*. Importantly, a wide niche breadth for a given environmental variable does not necessarily imply weak environmental control. Rather, it may reflect the influence of other unmeasured factors, such as biotic interactions or additional physicochemical variables not included in the present study. Therefore, this study provides a valuable case for investigating competition and coexistence among closely related phytoplankton species under natural environmental conditions.

## AUTHOR CONTRIBUTIONS

**Toshiya Katano:** Conceptualization (equal); formal analysis (equal); writing – original draft (lead). **Tomohiro Suematsu:** Investigation (lead). **Ayane Tanaka:** Investigation (supporting). **Saori Yasui‐Tamura:** Investigation (supporting). **Fuminori Hashihama:** Data curation (supporting); validation (supporting); writing – review and editing (supporting).

## Supporting information


**Figure S1.** Correlation of *Skeletonema* cell densities between light microscopic counts and qPCR estimations. The X‐axis of the upper panel represents total *Skeletonema*, while that of the lower panel represents *Skeletonema* excluding *S. menzelii* and *S. potamos* from the total.

**Figure S2.** Pairwise correlations among the five dominant *Skeletonema* species in Tokyo Bay.

**Table S1.** Information for *Skeletonema* strains used for the present study.

**Table S2.** Summary of calibration curve parameters for species‐specific real‐time PCR assays of *Skeletonema*.
