# Supplementary figures and images for: Seasonal succession and niche differentiation in Skeletonema species driven by temperature and salinity in inner Tokyo Bay
Source: J Phycol. 2026 Apr 24;62(3):931–42. doi: 10.1111/jpy.70168 (PMC13280773; doi:10.1111/jpy.70168)

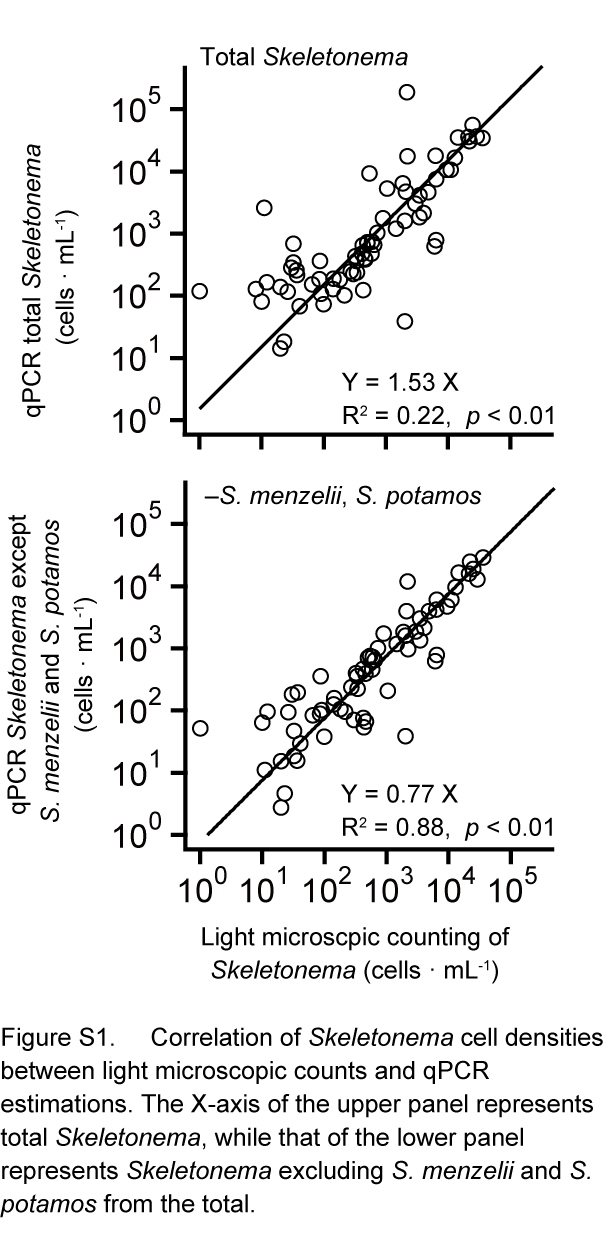

Supplement: Supplementary file 1 — Figure S1. Correlation of Skeletonema cell densities between light microscopic counts and qPCR estimations. The X‐axis of the upper panel represents total Skeletonema, while that of the lower panel represents Skeletonema excluding S. menzelii and S. potamos from the total. [file JPY-62-931-s001.png]

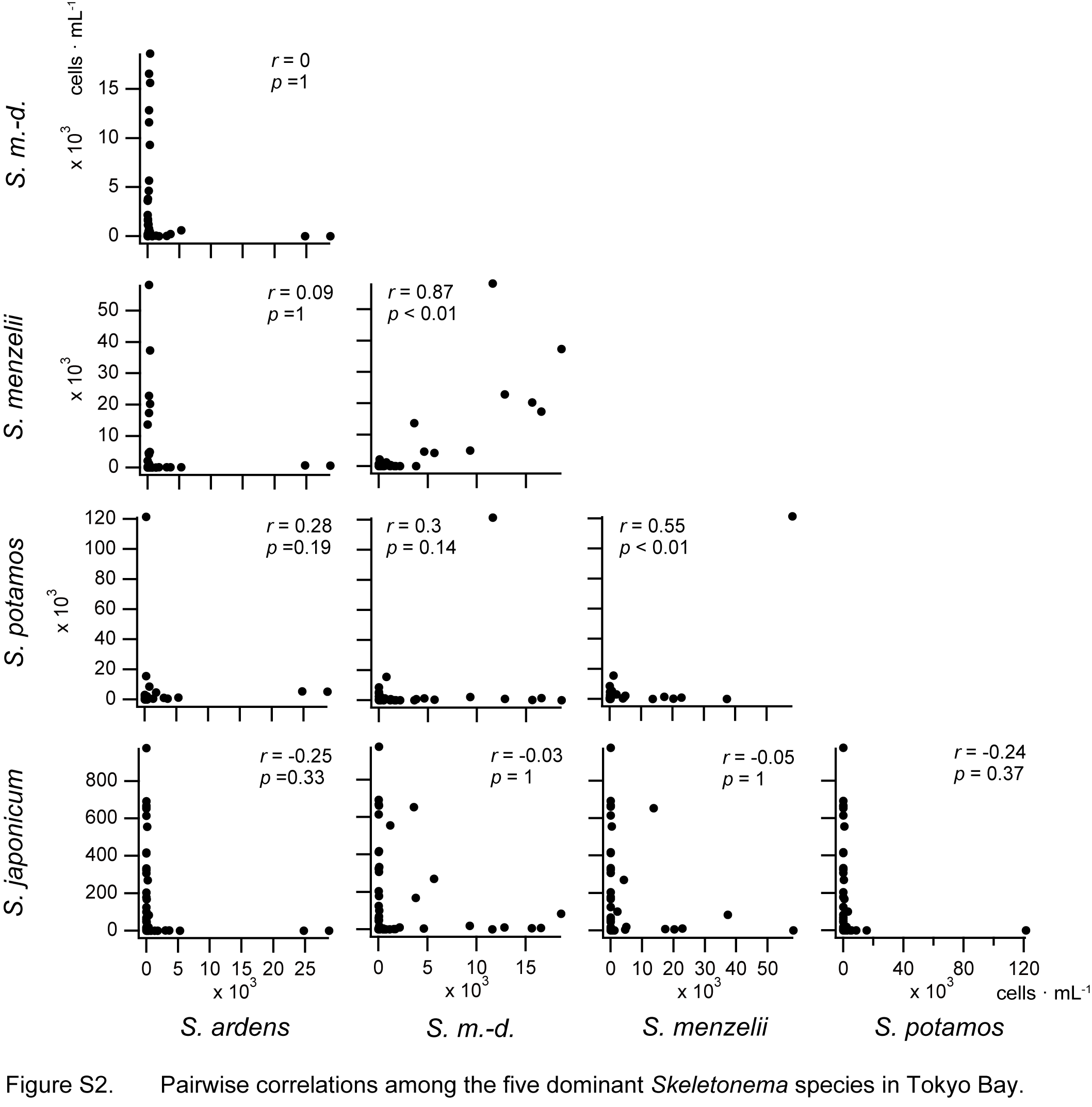

Supplement: Supplementary file 2 — Figure S2. Pairwise correlations among the five dominant Skeletonema species in Tokyo Bay. [file JPY-62-931-s002.png]
