# Supplementary material for: Seasonal succession and niche differentiation in Skeletonema species driven by temperature and salinity in inner Tokyo Bay
Source: J Phycol. 2026 Apr 24;62(3):931–42. doi: 10.1111/jpy.70168 (PMC13280773; doi:10.1111/jpy.70168)
Supplement: Supplementary file 3 — Table S1. Information for Skeletonema strains used for the present study. [file JPY-62-931-s004.docx]

| Table S1 Information for *Skeletonema* strains used for the present study. | | | | |  | |
| --- | --- | --- | --- | --- | --- | --- |
| Species | Strain | location of Isolation | Reference | Accession num. | |  |
| *S. ardens* | TKC092 | Tokyo Bay | Enjoji et al., 2019 | LC456866 | |  |
| *S. costatum* | TKC103 | Tokyo Bay | Enjoji et al, 2019 | LC456856 | |  |
| *S. dohrnii* | FDK033 | Dokai Bay | Yamada et al., 2010 | LC258378 | |  |
| *S. grevillei* | FON073 | Onagawa Bay | Enjoji et al., 2019 | LC258385 | |  |
| *S. japonicum* | TKC033 | Tokyo Bay | Enjoji et al., 2019 | LC456858 | |  |
| *S. menzelii* | DM08090730 | Dokai Bay | Yamada et al, 2010 | LC258392 | |  |
| *S. potamos* | P10#20 | Chikugo River | Enjoji et al., 2019 | LC258398 | |  |
| *S. pseudocostatum* | FDK225 | Dokai Bay | Yamada et al., 2010 | AB572833 | |  |
| *S. tropicum* | TKC156 | Ariake Sea | Enjoji et al., 2019 | LC456869 | |  |

References

Enjoji, N., Katano, T., Yoshinaka, Y., Furuoka, F., Ando, Y., Yamada, M., Hamasaki, T., Miyamura, E., Otsubo, M., & Yokoyama, K. (2019). Development of primer sets for a multiplex and qPCR assays targeting *Skeletonema* species and their application to field samples. *Journal of Oceanography*, 75, 319–334.

Yamada, M., Katsuki, E., Otsubo, M., Kawaguchi, M., Ichimi, K., Kaeriyama, H., Tada, K., & Harrison, P. J. (2010). Species diversity of genus *Skeletonema* (Bacillariophyceae) in the industrial harbor Dokai Bay, Japan. *Journal of Oceanography*, 66, 755–771.
