# Supplementary material for: Seasonal succession and niche differentiation in Skeletonema species driven by temperature and salinity in inner Tokyo Bay
Source: J Phycol. 2026 Apr 24;62(3):931–42. doi: 10.1111/jpy.70168 (PMC13280773; doi:10.1111/jpy.70168)
Supplement: Supplementary file 4 — Table S2. Summary of calibration curve parameters for species‐specific real‐time PCR assays of Skeletonema. [file JPY-62-931-s003.docx]

| Table S2 Summary of calibration curve parameters for species-specific real-time PCR assays of *Skeletonema*. | | | | | |
| --- | --- | --- | --- | --- | --- |
| Species | Slope | Efficiency | *R*^2^ | *y*-Intercept | No. of experiment |
| *S. ardens* | -3.74 ± 0.21 | 1.86 ± 0.06 | 1.00 ± 0.00 | 33.41 ± 1.47 | 4 |
| *S. costatum* | -3.75 ± 0.17 | 1.85 ± 0.05 | 1.00 ± 0.00 | 33.79 ± 0.87 | 5 |
| *S. dohrnii* | -4.09 ± 0.25 | 1.75 ± 0.06 | 0.99 ± 0.00 | 35.8 ± 1.05 | 6 |
| *S. grevillei* | -3.8305 | 1.82 | 1.00 | 30.51 | 1 |
| *S. japonicum* | -4.05 ± 0.22 | 1.77 ± 0.05 | 1.00 ± 0.00 | 33.49 ± 1.89 | 5 |
| *S. menzelii* | -3.78 ± 0.28 | 1.84 ± 0.07 | 0.99 ± 0.00 | 34.03 ± 0.97 | 5 |
| *S. potamos* | -3.73 ± 0.18 | 1.82 ± 0.06 | 0.99 ± 0.00 | 36.53 ± 1.87 | 4 |
| *S. pseudocostatum* | -3.8040 | 1.83 | 1.00 | 33.18 | 1 |
| *S. tropicum* | -3.7939 | 1.83 | 1.00 | 31.14 | 1 |
